# Saliva-derived components can enhance the performance of toluidine blue in photodynamic therapy

**DOI:** 10.3389/fphar.2025.1538520

**Published:** 2025-02-13

**Authors:** Nicolas Macri, Luana Mendonça Dias, Ana Claudia Pavarina, Walter L. Siqueira, Felipe Fornias Sperandio

**Affiliations:** ^1^ College of Dentistry, University of Saskatchewan, Saskatoon, SK, Canada; ^2^ Laboratory of Applied Microbiology Department of Dental Materials and Prosthodontics, School of Dentistry, Sao Paulo State University (UNESP), Araraquara, Brazil

**Keywords:** oral cancer (OC), photodynamic therapy, photosensitizer, saliva, oral pathology and oral medicine

## Abstract

**Introduction:**

Oral Squamous Cell Carcinoma (OSCC) is the most common type of head and neck cancer worldwide. Currently, the most common treatment for OSCC includes a combination of surgery, radiation, and chemotherapy. However, despite the advances made in therapeutic strategies, the prognosis for patients diagnosed with OSCC remains poor, especially at later stages, which emphasizes the need for a novel treatment approach. Photodynamic therapy (PDT) has been employed as stand-alone or adjuvant therapy for OSCC.

**Methods:**

This study investigated the potential of using salivary proteins such as histatin-5 (Hst5) or derived peptides (RR14, DR9/RR14) to perform histatin-mediated PDT. The current literature has shown that histatins have the capacity to increase cellular membrane permeability, which indicates a potential synergistic effect when combined with a photosensitive agent. Toluidine Blue O (TBO) was used as the photosensitizer (PS) singularly combined with salivary peptides RR14, DR9/RR14, and Hst5 protein, and experiments were conducted to assess its biocompatibility and photodynamic effects on human gingival fibroblasts (FGH) and oral squamous cell carcinoma (SCC-25) cell lines.

**Results:**

The results showed that TBO concentrations below 4 μg/mL were non-cytotoxic to FGH cells, whereas concentrations up to 8 μg/mL were non-cytotoxic to SCC-25 cells. Also, the presence of histatins did not modify the absorption spectrum or photobleaching of TBO, enabling consistent production of reactive oxygen species (ROS) over time and rendering it as a stable and suitable PS for PDT. Further experiments also showed that when TBO was combined with Hst5, the ROS production increased by 186% compared to TBO alone.

**Conclusion:**

Results suggest that the use of histatin-enhanced PS offer a promising alternative to conventional PDT, potentially improving its outcomes.

## Introduction

Oral Squamous Cell Carcinoma (OSCC) is the most common type of head and neck cancer worldwide, which includes a group of neoplasms most often affecting the lateral and ventral tongue, lips, and the floor of the mouth (Imbesi Bellantoni et al., 2023; Markopoulos, 2012). In 2020, of the 36 most common cancers worldwide, over 377,000 new cases of oral cancer were reported (Sung et al., 2021). According to the Canadian Cancer Society, it is estimated that by the end of 2024, 8,100 Canadians will be diagnosed with head and neck cancer, with 72% of those new cases being men (Canadian Cancer Society, 2024a). The 5-year mortality rate for stage 1 and 2 OSCC localized to the lip, tongue and floor of the mouth are 7%, 22%, and 25%, respectively (Canadian Cancer Society, 2024b). However, once the cancer progresses to stage 4, the 5-year mortality rates increases to 48%, 64%, and 80% for the lips, tongue, and floor of mouth respectively (Canadian Cancer Society, 2024b). Prominent risk factors for developing oral cancer include tobacco use, alcohol consumption, and potentially infections with viruses such as human papilloma virus (HPV), herpes group virus, adenovirus, and hepatitis C virus (Imbesi Bellantoni et al., 2023).

Currently, chemotherapy, radiation therapy, and surgery are the primary treatments given to patients with OSCC, with surgery being the most common course of action (Imbesi Bellantoni et al., 2023). To treat stages 1 and 2 of OSCC, a mixture of surgery and radiation therapy is often used (Markopoulos, 2012). However, for stage 3 and 4, chemotherapy is also used to manage the disease more aggressively, often in combination with surgery and radiation therapy, to control its spread (Markopoulos, 2012). A better prognosis is generally acknowledged to result from an early diagnosis (Bugshan and Farooq, 2020). For this reason, it is frequently advised that all new patients of physicians and dentists have a physical head and neck examination in order to detect and diagnose cancers early on, which can improve treatment outcomes and prognoses (Bugshan and Farooq, 2020).

In recent years, emerging therapies such as photodynamic therapy (PDT) are being explored for their potential in enhancing current cancer treatment outcomes. PDT makes use of a photosensitize molecule called a photosensitizer (PS), which is activated by a specific wavelength and intensity of light (Allison and Moghissi, 2013). Upon its activation, it can transfer its electrons to surrounding oxygen molecules to generate reactive oxygen species (ROS) such as singlet oxygen and hydroxyl radicals (Sperandio et al., 2013). When the amount of ROS exceeds the physiological range, they gain the potential to induce cell death by damaging the cell membrane, proteins, and DNA, ultimately leading to the destruction of the cells (Alvarez and Sevilla, 2024). Since the main impacted tissue is only the tissue that is exposed to light, PDT has several advantages over conventional cancer treatment methods (Mosaddad et al., 2023), including improved tumor selectivity and fewer side effects (Sperandio et al., 2013).

Human saliva contains a class of salivary proteins called histatins (Moffa et al., 2015). Some of these proteins include histatin-3 (Hst3) and histatin-5 (Hst5) which is a proteolytic fragment of Hst3 (Zolin et al., 2021). Both of these proteins are produced and secreted by the major salivary glands and are naturally found in human saliva (Zolin et al., 2021). Both Hst3 and Hst5 have demonstrated the ability to enhance cell permeability and be internalized to exert an antifungal effect on *Candida albicans*, resulting in the destruction of the cells (Zolin et al., 2021; Xu et al., 1999). Hst5 is known to promote membrane permeability in fungal cells, notably *Candida* albicans, by disturbing the ionic equilibrium consequently inducing an efflux of ions such as ATP and potassium (Xu et al., 1999; Gholami et al., 2022). This mechanism is mediated by electrostatic interactions between Hst5’s cationic residues and negatively charged phospholipids on the cell membrane (Xu et al., 1999; Gholami et al., 2022). Its internalization is energy-dependent and reliant on transmembrane potential, suggesting that it might leverage similar qualities in other cells, perhaps including tumor cells.

Currently, the impact of combining the photosensitizer with salivary proteins such as Hst5 on PDT is unknown. Given the demonstrated ability of Hst5 to interact with fungal cells, it is plausible that it could also interact with human cells, potentially augmenting the effects of PDT (Agostinis et al., 2011; Felgentrager et al., 2013). Based on this, it is hypothesized that histatin-mediated PDT could lead to improved outcomes than conventional PDT.

## Materials and methods

### Cell lines and cultures conditions

Human gingival fibroblasts (FGH) cells were grown in T75 flasks with media composed of 89% Dulbecco’s Modified Eagle Medium (DMEN), high glucose, no pyruvate (Gibco, Waltham, Massachusetts, United States), 10% fetal bovine serum (FBS), and 1% penicillin (antibiotic). Squamous cell carcinoma (SCC-25) cells were also used and grown in T75 flasks with media composed of FGH cell media and Hams Nutrient Mixture F12 (Gibco, Waltham, Massachusetts, United States) at a 1:1 ratio. Both cell lines were placed in an incubator at 37°C and 5% CO_2_ and the media was replaced every 2 days to remove debris. Before changing the media, the flasks were washed with phosphate buffered saline (PBS) 3 times before inserting fresh media into the flask.

### Cell viability analysis by alamarBlue™ following TBO exposure

To elucidate the biocompatibility of TBO with FGH and SCC-25 cell lines, a volume of 200 μL of cells were resuspended in their respective media, plated in 96-well plates, and incubated at 37°C with 5% CO_2_ for 24 h. Cells adhered to the bottom of the plate were washed with PBS and exposed to a range of TBO concentrations based in the current literature (Gholami et al., 2022) – 0.5, 1, 2, 4, 8, 16, 32, 64, 128, 256 μg/mL. After the PBS wash, 150 μL of fresh media was added into each well. Starting from the first sample column, 150 μL of TBO at 512 μg/mL was added and serially diluted across the plate, maintaining a final volume of 150 μL in each well. The control consisted of alamar Blue™ and cell media (2:15). The death control consisted of alamar Blue™ and cell media (2:15) as well as 1% Triton x-100. The sample wells consisted of alamar Blue™, cell media, and TBO (4:15:15). Afterwards, the sample solutions consisting of 12% alamar Blue™, 44% of respective cell media, and 44% TBO were incubated for 24 h at 37°C with 5% CO_2_, and read using BioTek Synergy HT Plate Reader. (Biotek Synergy HT, BioTek Instruments, Winooski, VT, United States; FL; excitation—530 nm; emission—590 nm). Two different occasions were performed containing quadruplicates. (n = 8/group). The results obtained were normalized and classified according to ISO 10993-5:2009 cytotoxicity guidelines.

### Cell viability analysis by alamar Blue™ following Hst5, RR14, and DR9/RR14 exposure

Protocol used was nearly identical to that of the cell viability analysis following TBO exposure, expect that cells were exposed to Hst5, RR14, and DR9/RR14 at varying concentrations that were based on the published literature (Moffa et al., 2015) of 32, 64, 128, 256, 512 μM. One occasion was performed containing duplicates. (n = 2/group). The results obtained were normalized and classified according to ISO 10993-5:2009 cytotoxicity guidelines.

### Absorbance spectrum analysis of TBO in the presence and absence of peptides

To elucidate the absorbance spectrum of TBO with and without peptides, a 96-well plate was used. Each well was filled to a volume of 150 μL and all reagents used were dissolved in Hanks’ balanced salt solution. Hst5, RR14, and DR9/RR14 were used at a concentration of 512 μM, and TBO was used at a concentration of 4 μg/mL (13.1 μM). The control wells contained Hanks’ balanced salt solution. Samples were divided into three groups (Imbesi Bellantoni et al., 2023): peptides (Hst5, RR14, and DR9/RR14) mixed with Hanks’ balanced salt solution in a 1:1 ratio (n = 2/group) (Markopoulos, 2012); TBO mixed with Hanks’ balanced salt solution in quadruplicate (n = 4); and (Sung et al., 2021) TBO combined with peptides in a 1:1 ratio (n = 2/group). 96-well plate was read immediately after its preparation using BioTek Synergy HT Plate Reader. (Biotek Synergy HT, BioTek Instruments, Winooski, VT, United States; Abs; range – 300 nm–800 nm).

### Red light fluence analysis of TBO in the presence and absence of peptides

To calculate the dosage of red light needed to produce the maximum amount of ROS, a 96-well plate was used. Each well was filled to a volume of 150 μL and all reagents used were dissolved in Hanks’ balanced salt solution (HBSS). Hst5, RR14, and DR9/RR14 were used at a concentration of 512 μM, and TBO was used at a concentration of 4 μg/mL. The control wells consisted of HBSS (n = 6). The samples were divided into 3 groups. The first group contained duplicates of peptides (Hst5, RR14, and DR9/RR14) and HBSS at a 1:1 ratio (n = 2/peptide). The second group contained samples of TBO dissolved in HBSS at a 1:1 ratio (n = 8). The third group contained duplicates of TBO and peptides at a 1:1 ratio (n = 2/peptide). 96-well plate was placed in the BioTable (Bio Table RGB, São Carlos, Brazil) immediately after its preparation for 0.38 min (0.5 J/cm^2^). Following this, the 96-well plate was read using BioTek Synergy HT Plate Reader. (Biotek Synergy HT, BioTek Instruments, Winooski, VT, United States; FL; excitation—485 nm; emission—528 nm). This was repeated for the other timepoints that correspond with the doses shown in Table 1.

**TABLE 1:** Time (min) required to reach specific energy densities with BioTable.

Dosage of red light (J/cm^2^)	Exposure time (min)
0.5	0.38
10	7.58
15	11.36
20	15.15
25	18.94
30	22.73
35	26.31
40	30.30
45	34.09
50	37.88

### Photobleaching of TBO in the presence and absence of peptides

To monitor the degradation of TBO with and without peptides over time, a 96-well plate was used. Each well was filled to a volume of 150 μL and all reagents used were dissolved in Hanks’ balanced salt solution. Hst5, RR14, and DR9/RR14 were used at a concentration of 512 μM, and TBO was used at a concentration of 4 μg/mL. The control wells consisted of Hanks’ balanced salt solution (n = 6). The samples were divided into 3 groups. The first group contained duplicates of peptides (Hst5, RR14, and DR9/RR14) and Hanks’ balanced salt solution at a 1:1 ratio (n = 2/group). The second group contained samples of TBO dissolved in Hanks’ balanced salt solution at a 1:1 ratio (n = 8). The third group contained duplicates of TBO and peptides at a 1:1 ratio (n = 2/group). 96-well plate was read immediately after its preparation using BioTek Synergy HT Plate Reader. (Biotek Synergy HT, BioTek Instruments, Winooski, VT, United States; Abs; 630 nm) to establish the baseline amount of TBO at 0 min 96-well plate was placed in BioTable (Bio Table RGB, São Carlos, Brazil) and subsequent readings occurred at the 5 min, 10 min, 15 min, 20 min, 30 min, and 40 min timepoints.

### Statistical analysis

Data normality was assessed using the Shapiro-Wilk test, while homogeneity of variances was evaluated with Levene’s test. For data following a normal distribution, one-way ANOVA was applied. Homoscedastic data were analyzed using Tukey’s *post hoc* test (Cell Viability Analysis), whereas heteroscedastic data were subjected to Welch’s correction, followed by the Games-Howell *post hoc* test. When two fixed factors were identified, two-way ANOVA with Bonferroni correction was used (ROS Production and Absorbance Spectrum Analysis). For non-normally distributed data, the Kruskal–Wallis test was employed, followed by Dunn’s *post hoc* test (Red Light Fluence and Photobleaching). A significance level of 5% was set for all statistical analyses. All data analyses were performed using IBM SPSS Statistics 30.0 software, and graphs were generated with GraphPad Prism.

## Results

**FIGURE 1:**
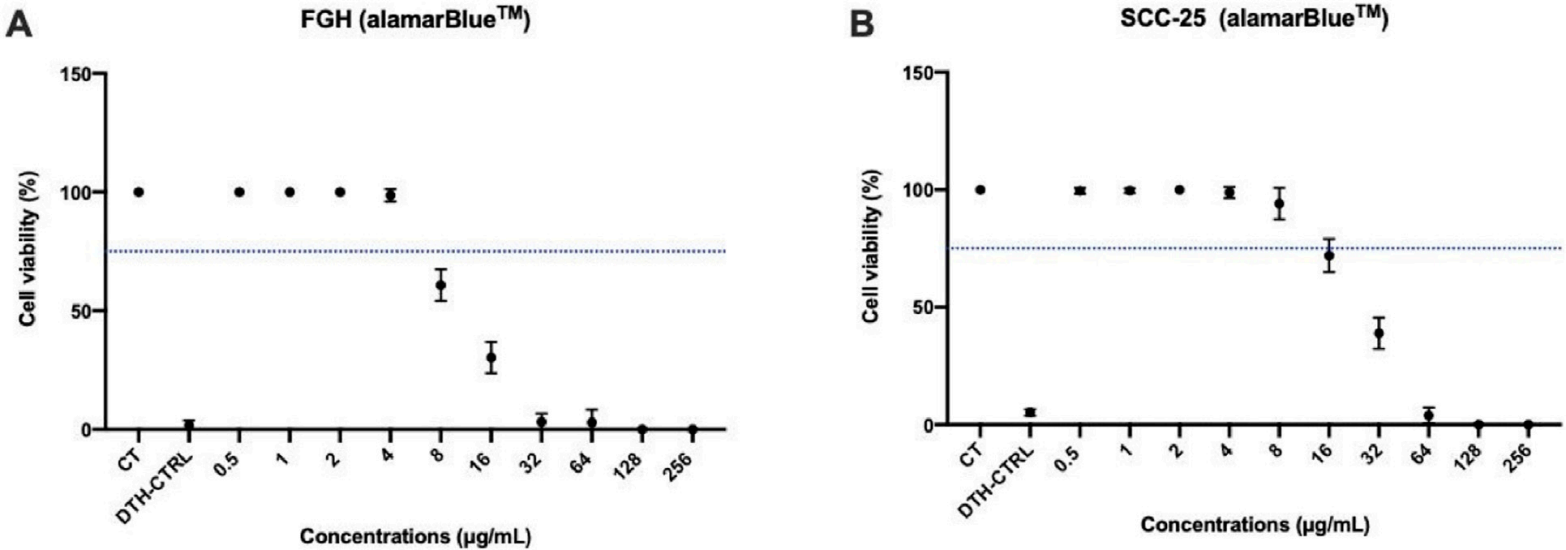
Effects of TBO on cell viability. Mean and 95% confidence interval of Cell Viability (%) of Cells FGH **(A)** and SCC-25 **(B)** treated with increasing concentrations of TBO and measured by alamarBlue™. Concentrations of TBO that result in a viability greater than 75% was deemed to be non-cytotoxic to the cells. The blue dotted horizontal line indicates the 75% viability threshold. **(A)** Concentrations of TBO *versus* FGH cells. **(B)** Concentrations of TBO *versus* SCC-25 cells. CT = Control, DTH-CTRL = death control. Points: data means. Error bars: minimum and maximum values. Points: data means. Error bars: minimum and maximum values. The non-intersection of the error bars denotes a difference according to the 95% confidence interval validated by Tukey test *post hoc* (ANOVA-one way) (p < 0.05).

FGH and SCC-25 cells were exposed to increasing concentrations of TBO. The concentrations started at 0.5 μg/mL and were doubled until 256 μg/mL. Cell viability was measured as a percent in comparison to the control. Figure 1 shows the cell viability graphs with a blue dotted line which represents a 25% reduction in viability. According to the ISO 10993-5:2009 guidelines, any concentration of TBO that results in a reduction of viability less than 25% is considered to be non-cytotoxic.

For the FGH cell line, concentrations of TBO ≤4 μg/mL was deemed to be non-cytotoxic to the cells (p > 0.05). Also, for the SCC-25 cell line, any concentrations of TBO ≤8 μg/mL was deemed to be non-cytotoxic to the cells (p > 0.05). For both cells lines, concentrations ranging from 0.5 μg/mL to 4 μg/mL were consistently measured near 100% viability compared to the control, meaning that these concentrations of TBO resulted in little to no cell death after 24 h of exposure to TBO.

For subsequent experiments, a single concentration of TBO was chosen for both cell lines to maintain consistency. To simplify the process, 4 μg/mL (p = 0.07) was selected as the standardized concentration for all experiments. This concentration was the maximum that FGH cells could tolerate without exhibiting cytotoxicity. Although SCC-25 cells could tolerate up to 8 μg/mL (p = 0.059), using 4 μg/mL ensured a uniform approach across both cell lines.

**FIGURE 2:**
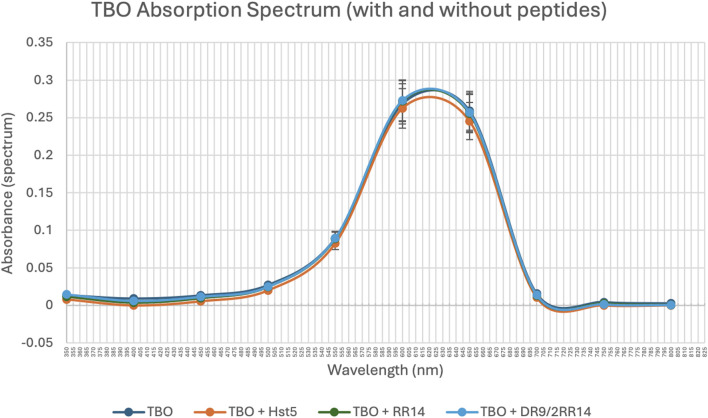
TBO Absorption Spectrum in the Presence and Absence of Peptides (spectrum). The absorption spectrum of TBO (4 μg/mL) was measured with and without the addition of peptides: Hst5 (512 μM), RR14 (512 μM), and DR9/2RR14 (512 μM). The absorption spectrum for each sample was recorded using a spectrophotometer. Points: data means. Error bars: minimum and maximum values. The non-intersection of the error bars denotes a difference according to the 95% confidence interval validated by Bonferroni test *post hoc* (ANOVA-two way) (p < 0.05).

After choosing a TBO concentration of 4 μg/mL, it was imperative to analyze the absorbance spectrum of TBO in the presence and absence of peptides to verify that peptides did not modify TBO’s absorbance properties. When peptides were not present, TBO showed a maximum absorption at around 630 nm (580 nm: p = 0.034 compared to the baseline/650 nm: p = 0.004 compared to the baseline), which matches the documented maximum absorption for TBO (Figure 2). The inclusion of peptides did not result in any notable alteration in the absorbance spectrum that impacted the maximum absorbance of TBO. Therefore, as the peptides did not modify the absorbance spectrum, a wavelength of 630 nm can be employed for photodynamic therapy (PDT) with or without peptides to guarantee optimal activation of TBO.

**FIGURE 3:**
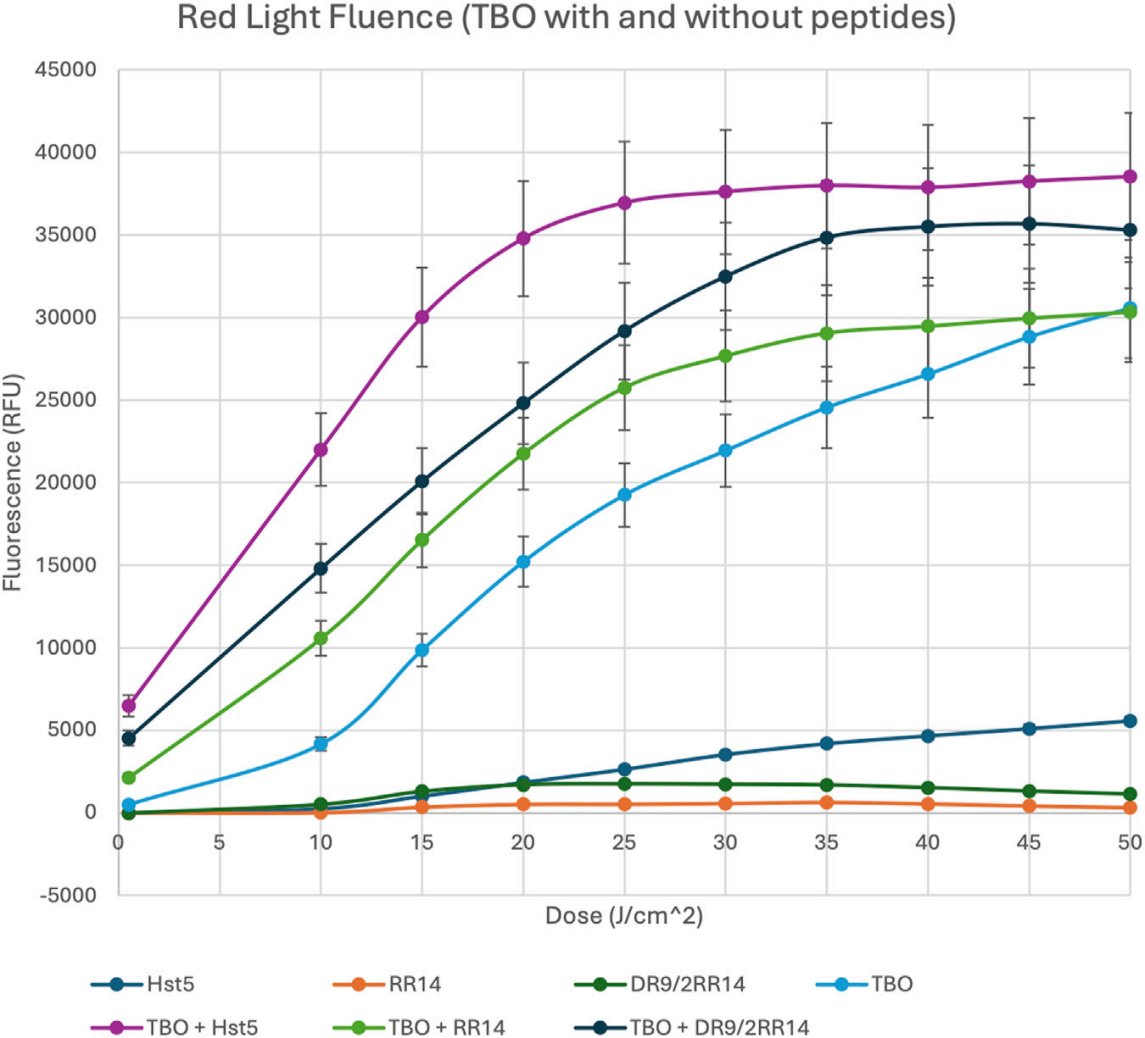
Red Light Fluence of TBO in the Prescence and Absence of Peptides. H2DCFDA (2′,7′-dichlorofluorescin diacetate) fluorescent probe used to bind and detect ROS such as superoxide anions, hydrogen peroxide, and hydroxyl radicals. Samples exposed to red light (630 nm) over time for increasing amount of dose and ROS were measured in a spectrophotometer at an excitation of 485 nm and an emission of 528 nm. Points: data means. Error bars: minimum and maximum values. The non-intersection of the error bars denotes a difference according to the 95% confidence interval validated by Dunn’s *post hoc* test (Kruskal–Wallis) (p < 0.05).

**FIGURE 4:**
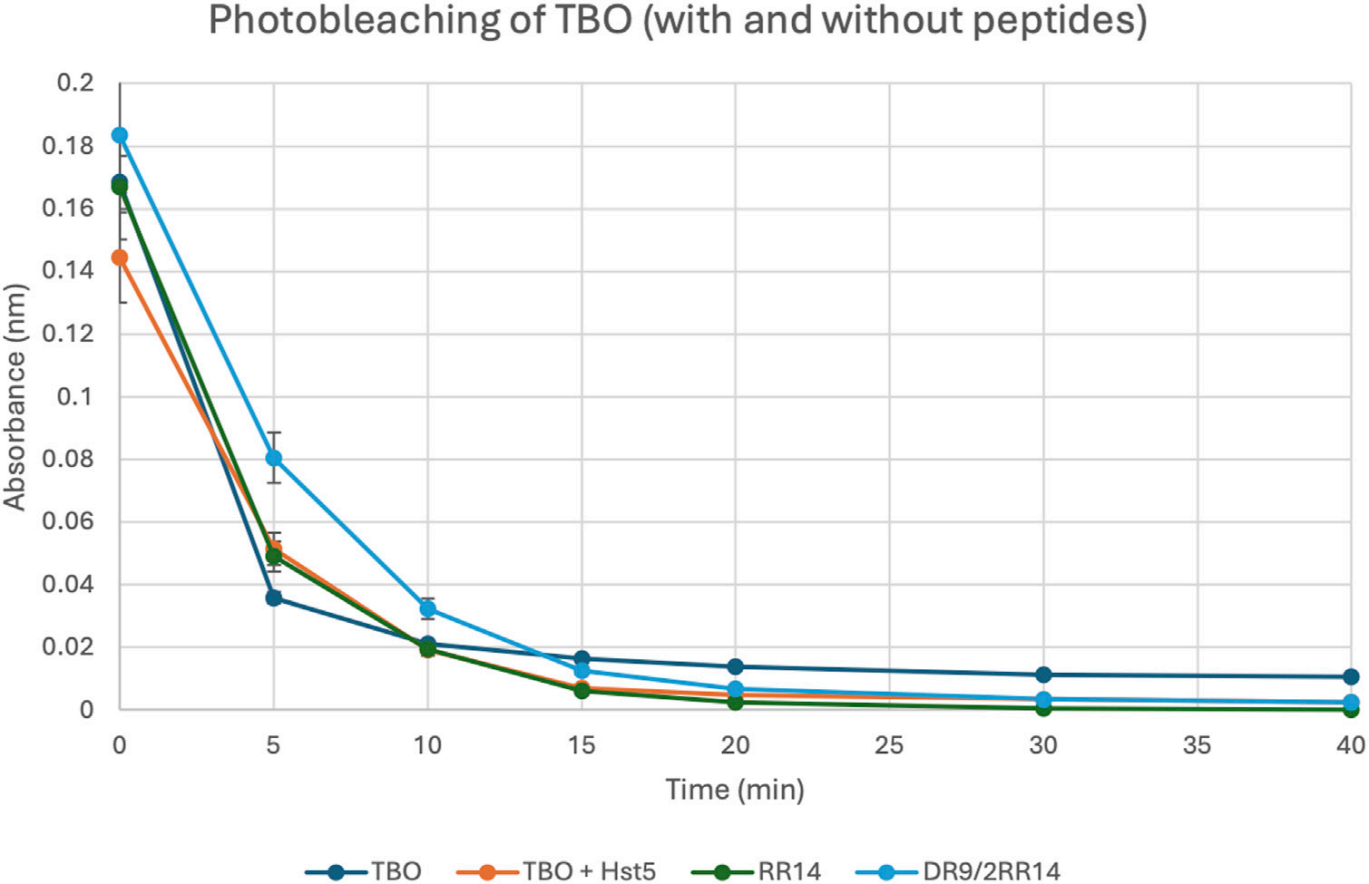
TBO Photobleaching in the Presence and Absence of Peptides. The breakdown of TBO (4 μg/mL) was measured with and without the addition of peptides: Hst5 (512 μM), RR14 (512 μM), and DR9/2RR14 (512 μM). The absorption rate of each well was recorded using a spectrophotometer at 630 nm. (Biotek Synergy HT, BioTek Instruments, Winooski, VT, United States; Abs; 630 nm). Points: data means. Error bars: minimum and maximum values. The non-intersection of the error bars denotes a difference according to the 95% confidence interval validated by Dunn’s *post hoc* test (Kruskal–Wallis) (p < 0.05).

After it was confirmed that the peptides did not interact with TBO in a way that shifted the absorbance, the ROS production was then monitored over time using a probe (2′,7′-dichlorofluorescin diacetate). As the dose of light increased over time, the ROS production also increased (p = 0.032); also, at around a dose of 35 J/cm^2^, the ROS production reaches a plateau (Figure 3). This dose corresponds with a time of 26.31 min (Table 1), which is in accordance with the photobleaching of TBO where TBO breakdown was monitored over time. The less TBO available, the slower the increase in ROS production from one dose to the next (Figures 3, 4). The graph clearly shows that when proteins such as Hst5, RR14, and DR9/2RR14 were combined with TBO, the ROS production increased significantly (Figure 3). At a dose of 15 J/cm2, equivalent to a time of 11.36 min, the combination of TBO + Hst5 resulted in an average increase of 185.7% in ROS production compared to TBO alone (p = 0.023). For TBO + RR14, and TBO + DR9/2RR14, the average increases in ROS production were 61.3%, and 94.0% (p = 0.034), respectively (Figure 3). The final dose measured was 50 J/cm^2^, equivalent to a time of 37.88 min (Table 1). At this dose, the combination of TBO with Hst5 still resulted in a greater amount of ROS production and resulted in an average increase of 22.7% in ROS production compared to TBO alone (p = 0.043) (Figure 3). For TBO + RR14, and TBO + DR9/2RR14, the average increases in ROS production at a dose of 50 J/cm^2^ were −0.7% (p = 0.09), and 13.5% (p = 0.039) respectively (Figure 3). The results clearly demonstrate that the combination of Hst5 and TBO significantly enhances the generation of ROS at all doses and time points. However, the extent of this increase is particularly pronounced at lower doses and time points.

After establishing that the combination of TBO and Hst5 greatly increased ROS generation, photobleaching of TBO was observed throughout time to assess its degradation after photodynamic treatment (PDT) exposure (Figure 4). The results showed no significant changes in the photobleaching kinetics of TBO alone vs. TBO coupled with peptides such as Hst5, RR14, and DR9/2RR14. All TBO-containing samples were exposed to 630 nm red light for 40 min, and the absorbance was measured with a spectrophotometer. The near identical photobleaching patterns observed imply that the peptides do not inhibit TBO photodegradation during PDT.

As shown in Figure 4, TBO alone exhibited rapid photobleaching within the first 5–10 min of light exposure, with absorbance decreasing sharply and stabilizing at minimal levels thereafter. This pattern was consistent for TBO combined with peptides, including Hst5, RR14, and DR9/2RR14. Importantly, no significant differences were observed in the photobleaching profiles between TBO alone and TBO in the presence of peptides, indicating that the peptides did not interfere with the photodegradation of TBO.

## Discussion

There are known limitations with conventional therapies (surgery, chemotherapy and radiotherapy), particularly in advanced stages of OSCC, where poor prognosis is often recorded, and side effects may outweigh benefits. The photodynamic mechanism of PDT, which localizes the cytotoxic effect to light-exposed tissues, offers a targeted approach with fewer systemic side effects. This study aimed to assess the potential of histatin-mediated photosensitizers as promising photosensitive agents for PDT, with specific regards to antineoplastic PDT, and as a possible future alternative treatment for OSCC.

An ideal PS for antineoplastic PDT should have a high absorption peak between 600 and 800 nm given that longer wavelengths (>800 nm) do not provide enough energy to yield significant generation of ROS, i.e., excitation of molecular oxygen to its singlet state (Agostinis et al., 2011). Therefore, checking the absorption peak of TBO to investigate if either hist5 or related peptides would influence its absorption spectrum was one of our priorities. In fact, none of the added saliva-derived peptides/protein was able to modify TBO’s absorbance properties; TBO alone showed a maximum absorption at around 630 nm, which matches the documented maximum and ideal absorption for this PS, while the inclusion of the added components did not result in any notable alteration in the absorbance spectrum that impacted the maximum absorbance of TBO. Therefore, as the added components did not modify the absorbance spectrum, a wavelength of 630 nm can be employed for histatin-mediated photodynamic therapy (PDT) to guarantee optimal activation and ROS generation.

Also, the properties of a PS can change after light exposure, impacting their absorption and fluorescence characteristics, implying in photodegradation and/or the formation of photoproducts, thus pointing to reduced photostability (Felgentrager et al., 2013; Ferreira et al., 2008; Kiesslich et al., 2013); these alterations are often reflected in the emergence of new absorption bands within the PS’s specific absorption spectrum (Ericson et al., 2003). Monitoring degradation can be achieved by observing a decrease in the maximum absorption peak of the PS (Kiesslich et al., 2013), as performed herein. Successful inactivation of microorganisms or tumors will be incomplete if the PS is bleached too quickly and the concentration of nondegraded photosensitizer falls below the minimum inhibitory threshold during illumination (Kiesslich et al., 2013; Moan, 1986). Conversely, photobleaching can be beneficial in reducing overall skin photosensitivity, a common side effect in patients undergoing PDT (Agostinis et al., 2011; Roberts et al., 1989).

As withdrawn from our results, photobleaching of TBO was either unchanged or positively affected by adding Hst5 or salivary peptides, especially RR14, as photodegradation was initially retarded at initial time points (5 and 10 min). This would signify a potential increase in the tumor concentration of TBO, especially at early time points, which are the desired timeframe when designing a PDT protocol (as long as a clinically relevant range is employed, relatively shorter irradiation time is preferred over longer periods for practical applications and antitumor activity, as it mainly conserves tissue oxygenation and subsequent higher ROS generation (Carigga Gutierrez et al., 2022). In addition, at later time points (past 15 min of irradiation), photodegradation was significantly augmented for all groups with added protein/peptides, which is another advantage of histatin-mediated PDT as it helps diminish skin/mucosal photosensitivity.

Ultimately, our results provide encouraging evidence that the employed salivary peptides, and especially Hst5, may significantly enhance the efficacy of PDT by amplifying ROS production when combined with TBO. The significant amplification in ROS production when TBO was combined with Hst5 aligns with the hypothesis that histatins could augment PDT outcomes by enhancing cell membrane permeability and potentially improving photosensitizer uptake, as observed in fungal cell studies (Zolin et al., 2021; Xu et al., 1999). The ROS generation curves were successful as expected, reaching a plateau at the very end of the experiment. In fact, photobleaching and limited availability of oxygen at higher doses may contribute to the well-known plateau phenomenon that was observed (Sperandio et al., 2013). In addition, the aggregation of certain PS, i.e., Rose Bengal, may eventually restrict light-induced ROS production by means of self-quenching at certain fluences (Liu et al., 2013; Tang et al., 2021).

In fact, significant knowledge gaps exist regarding the mechanisms through which PDT improves cancer cell permeability, particularly the involvement of critical signaling pathways and how these pathways are modulated by the complex interplay of PDT dosimetry parameters (Carigga Gutierrez et al., 2022). These gaps are especially relevant as PDT’s ability to enhance permeability may directly influence the uptake and efficacy of therapeutic agents, such as photosensitizers combined with adjunctive agents like histatins. Understanding the specific cellular and molecular events triggered by PDT, including membrane dynamics, oxidative stress responses, and intracellular signaling is essential to optimize its application (Castano et al., 2004).

Furthermore, these processes may be dose-dependent, necessitating detailed investigation into the optimal light fluence, duration, and concentration of therapeutic agents to achieve maximal permeability without compromising cell selectivity or safety (Carigga Gutierrez et al., 2022). By addressing these knowledge gaps, future studies could significantly enhance the role of PDT as an adjuvant approach, particularly for therapies targeting tumors with otherwise poor permeability. This priming effect could broaden the eligibility of cancer patients for life-extending treatments, including those who are currently excluded due to tumor microenvironment barriers or systemic therapy limitations (Araki et al., 2015; Huang et al., 2018; Luo et al., 2019; Luo et al., 2016). In our study, while we demonstrate the potential of histatin-mediated PDT, however further exploration is necessary to elucidate these underlying mechanisms and optimize treatment protocols.

Consistent with previous literature (Rocha et al., 2023), TBO alone generated ROS at cytotoxic levels in OSCC cells without impacting normal human gingival fibroblasts (FGH) at concentrations of up to 4 μg/mL, while SCC-25 cells tolerated up to 8 μg/mL, ensuring biocompatibility within effective PDT doses. The enhancement observed with Hst5 was especially notable, with ROS production increasing by 186% compared to TBO alone, underscoring the potential of Hst5 as a synergistic agent in PDT. Future studies should address whether molecular integration of the two components (PS + Hist) should provide enhanced benefits as opposed to media combination as employed herein.

Regardless, our findings suggest that the combination of TBO and Hst5 in PDT could increase the therapeutic margin by enabling greater cytotoxicity in malignant cells while minimizing damage to surrounding healthy tissues, given that the employment of Hst5 yields a much higher production of ROS at early irradiation periods, therefore leading to more efficacious cell inactivation with lesser tissue damage given diminished time of PS contact with the targeted tissue.

The results contribute valuable insights toward developing PDT protocols that incorporate histatins as adjunctive agents, potentially adding to the plethora of available PS for OSCC PDT. Extrapolating from these data, the investigated approach could be a particularly viable alternative for early-stage OSCC, where targeted PDT could limit disease progression from early premalignant lesions (oral dysplasia) towards carcinoma *in situ* or truly invasive OSCC, ultimately improving long-term survival outcomes. Notably, PDT has demonstrated efficacy in treating other premalignant conditions, such as Barrett’s esophagus (Aebisher et al., 2024; Sanchez et al., 2010), and has shown promise as an alternative therapy for oral potentially malignant disorders (Jerjes et al., 2011; Narahara et al., 2023). Further studies are warranted to investigate the optimal Hst5 dosing and TBO-light exposure parameters in *vivo* settings to maximize OSCC cell death while maintaining a favorable safety profile of the surrounding viable tissue.

## Data Availability

Datasets are available on request: The raw data supporting the conclusions of this article will be made available by the authors, without undue reservation.

## References

[B1] Aebisher D. Rogoz K. Mysliwiec A. Dynarowicz K. Wiench R. Cieslar G. (2024). The use of photodynamic therapy in medical practice. Front. Oncol. 14, 1373263. 10.3389/fonc.2024.1373263 38803535 PMC11129581

[B2] Agostinis P. Berg K. Cengel K. A. Foster T. H. Girotti A. W. Gollnick S. O. (2011). Photodynamic therapy of cancer: an update. CA Cancer J. Clin. 61 (4), 250–281. 10.3322/caac.20114 21617154 PMC3209659

[B3] Allison R. R. Moghissi K. (2013). Photodynamic therapy (PDT): PDT mechanisms. Clin. Endosc. 46 (1), 24–29. 10.5946/ce.2013.46.1.24 23422955 PMC3572346

[B4] Alvarez N. Sevilla A. (2024). Current advances in photodynamic therapy (PDT) and the future potential of PDT-combinatorial cancer therapies. Int. J. Mol. Sci. 25 (2), 1023. 10.3390/ijms25021023 38256096 PMC10815790

[B5] Araki T. Ogawara K. Suzuki H. Kawai R. Watanabe T. Ono T. (2015). Augmented EPR effect by photo-triggered tumor vascular treatment improved therapeutic efficacy of liposomal paclitaxel in mice bearing tumors with low permeable vasculature. J. Control Release 200, 106–114. 10.1016/j.jconrel.2014.12.038 25553829

[B6] Bugshan A. Farooq I. (2020). Oral squamous cell carcinoma: metastasis, potentially associated malignant disorders, etiology and recent advancements in diagnosis. F1000Res 9, 229. 10.12688/f1000research.22941.1 32399208 PMC7194458

[B7] Canadian Cancer Society (2024a). Available at: https://cancer.ca/en/cancer-information/cancer-types/oral/statistics (Accessed November 28, 2024).

[B8] Canadian Cancer Society (2024b). Available at: https://cancer.ca/en/cancer-information/cancer-types/oral/prognosis-and-survival (Accessed November 28, 2024).

[B9] Carigga Gutierrez N. M. Pujol-Sole N. Arifi Q. Coll J. L. le Clainche T. Broekgaarden M. (2022). Increasing cancer permeability by photodynamic priming: from microenvironment to mechanotransduction signaling. Cancer Metastasis Rev. 41 (4), 899–934. 10.1007/s10555-022-10064-0 36155874

[B10] Castano A. P. Demidova T. N. Hamblin M. R. (2004). Mechanisms in photodynamic therapy: part one-photosensitizers, photochemistry and cellular localization. Photodiagnosis Photodyn. Ther. 1 (4), 279–293. 10.1016/S1572-1000(05)00007-4 25048432 PMC4108220

[B11] Ericson M. B. Grapengiesser S. Gudmundson F. Wennberg A. M. Larko O. Moan J. (2003). A spectroscopic study of the photobleaching of protoporphyrin IX in solution. Lasers Med. Sci. 18 (1), 56–62. 10.1007/s10103-002-0254-2 12627275

[B12] Felgentrager A. Maisch T. Dobler D. Spath A. (2013). Hydrogen bond acceptors and additional cationic charges in methylene blue derivatives: photophysics and antimicrobial efficiency. Biomed. Res. Int. 2013, 482167. 10.1155/2013/482167 23509728 PMC3591237

[B13] Ferreira J. M. P. Kurachi C. Sibata C. Allison R. R. Bagnato V. S. (2008). Photostability of different chlorine photosensitizers. Laser Phys. Lett. 5 (2), 156–161. 10.1002/lapl.200710099

[B14] Gholami L. Shahabi S. Jazaeri M. Hadilou M. Fekrazad R. (2022). Clinical applications of antimicrobial photodynamic therapy in dentistry. Front. Microbiol. 13, 1020995. 10.3389/fmicb.2022.1020995 36687594 PMC9850114

[B15] Huang H. C. Rizvi I. Liu J. Anbil S. Kalra A. Lee H. (2018). Photodynamic priming mitigates chemotherapeutic selection pressures and improves drug delivery. Cancer Res. 78 (2), 558–571. 10.1158/0008-5472.CAN-17-1700 29187403 PMC5771811

[B16] Imbesi Bellantoni M. Picciolo G. Pirrotta I. Irrera N. Vaccaro M. Vaccaro F. (2023). Oral cavity squamous cell carcinoma: an update of the pharmacological treatment. Biomedicines 11 (4), 1112. 10.3390/biomedicines11041112 37189730 PMC10135659

[B17] Jerjes W. Upile T. Hamdoon Z. Mosse C. A. Akram S. Hopper C. (2011). Photodynamic therapy outcome for oral dysplasia. Lasers Surg. Med. 43 (3), 192–199. 10.1002/lsm.21036 21412802

[B18] Kiesslich T. Gollmer A. Maisch T. Berneburg M. Plaetzer K. (2013). A comprehensive tutorial on *in vitro* characterization of new photosensitizers for photodynamic antitumor therapy and photodynamic inactivation of microorganisms. Biomed. Res. Int. 2013, 840417. 10.1155/2013/840417 23762860 PMC3671303

[B19] Liu K. Liu Y. Yao Y. Yuan H. Wang S. Wang Z. (2013). Supramolecular photosensitizers with enhanced antibacterial efficiency. Angew. Chem. Int. Ed. Engl. 52 (32), 8285–8289. 10.1002/anie.201303387 23804550

[B20] Luo D. Carter K. A. Molins E. A. G. Straubinger N. L. Geng J. Shao S. (2019). Pharmacokinetics and pharmacodynamics of liposomal chemophototherapy with short drug-light intervals. J. Control Release 297, 39–47. 10.1016/j.jconrel.2019.01.030 30684512 PMC6399029

[B21] Luo D. Carter K. A. Razi A. Geng J. Shao S. Giraldo D. (2016). Doxorubicin encapsulated in stealth liposomes conferred with light-triggered drug release. Biomaterials 75, 193–202. 10.1016/j.biomaterials.2015.10.027 26513413 PMC4644481

[B22] Markopoulos A. K. (2012). Current aspects on oral squamous cell carcinoma. Open Dent. J. 6, 126–130. 10.2174/1874210601206010126 22930665 PMC3428647

[B23] Moan J. (1986). Effect of bleaching of porphyrin sensitizers during photodynamic therapy. Cancer Lett. 33 (1), 45–53. 10.1016/0304-3835(86)90100-x 2945634

[B24] Moffa E. B. Machado M. A. Mussi M. C. Xiao Y. Garrido S. S. Giampaolo E. T. (2015). *In vitro* identification of histatin 5 salivary complexes. PLoS One 10 (11), e0142517. 10.1371/journal.pone.0142517 26544073 PMC4636238

[B25] Mosaddad S. A. Namanloo R. A. Aghili S. S. Maskani P. Alam M. Abbasi K. (2023). Photodynamic therapy in oral cancer: a review of clinical studies. Med. Oncol. 40 (3), 91. 10.1007/s12032-023-01949-3 36749489

[B26] Narahara S. Ikeda H. Ogata K. Shido R. Asahina I. Ohba S. (2023). Long-term effect of photodynamic therapy on oral squamous cell carcinoma and epithelial dysplasia. Photodiagnosis Photodyn. Ther. 41, 103246. 10.1016/j.pdpdt.2022.103246 36535598

[B27] Roberts W. G. Smith K. M. McCullough J. L. Berns M. W. (1989). Skin photosensitivity and photodestruction of several potential photodynamic sensitizers. Photochem Photobiol. 49 (4), 431–438. 10.1111/j.1751-1097.1989.tb09191.x 2727082

[B28] Rocha E. Bomfim L. Junior S. Santos G. Meira C. Soares M. (2023). Photodynamic therapy with an association of methylene blue and toluidine blue promoted a synergic effect against oral squamous cell carcinoma. Cancers (Basel) 15 (23), 5509. 10.3390/cancers15235509 38067213 PMC10705520

[B29] Sanchez A. Reza M. Blasco J. A. Callejo D. (2010). Effectiveness, safety, and cost-effectiveness of photodynamic therapy in Barrett's esophagus: a systematic review. Dis. Esophagus 23 (8), 633–640. 10.1111/j.1442-2050.2010.01078.x 20545970

[B30] Sperandio F. F. Sharma S. K. Wang M. Jeon S. Huang Y. Y. Dai T. (2013). Photoinduced electron-transfer mechanisms for radical-enhanced photodynamic therapy mediated by water-soluble decacationic C₇₀ and C₈₄O₂ Fullerene Derivatives. Nanomedicine 9 (4), 570–579. 10.1016/j.nano.2012.09.005 23117043 PMC3582824

[B31] Sung H. Ferlay J. Siegel R. L. Laversanne M. Soerjomataram I. Jemal A. (2021). Global cancer Statistics 2020: GLOBOCAN estimates of incidence and mortality worldwide for 36 cancers in 185 countries. CA Cancer J. Clin. 71 (3), 209–249. 10.3322/caac.21660 33538338

[B32] Tang P. El-Moghazy A. Y. Ji B. Nitin N. Sun G. (2021). Unique “posture” of rose Bengal for fabricating personal protective equipment with enhanced daylight-induced biocidal efficiency. Mater Adv. 2 (11), 3569–3578. 10.1039/d1ma00100k 34179787 PMC8186280

[B33] Xu Y. Ambudkar I. Yamagishi H. Swaim W. Walsh T. J. O'Connell B. C. (1999). Histatin 3-mediated killing of Candida albicans: effect of extracellular salt concentration on binding and internalization. Antimicrob. Agents Chemother. 43 (9), 2256–2262. 10.1128/AAC.43.9.2256 10471575 PMC89457

[B34] Zolin G. V. S. Fonseca F. H. D. Zambom C. R. Garrido S. S. (2021). Histatin 5 metallopeptides and their potential against Candida albicans pathogenicity and drug resistance. Biomolecules 11 (8), 1209. 10.3390/biom11081209 34439875 PMC8391865

